# Integrative transcriptomic profiling of tumor patients in surgical ICU: identifying prognostic immune signatures

**DOI:** 10.3389/fmolb.2026.1776416

**Published:** 2026-06-23

**Authors:** Changjin Chen, Hengquan Zhong, Songmao Ouyang, Jiying Lai, Chaoyu Wu

**Affiliations:** Department of Critical Care Medicine, The First Affiliated Hospital of Gannan Medical University, Ganzhou, Jiangxi, China

**Keywords:** immune signatures, prognostic biomarkers, surgical intensive care unit, transcriptomics, tumor immunity

## Abstract

Prognostic assessment of tumor patients in the surgical intensive care unit (SICU) remains challenging due to the complex interplay between systemic inflammation and immune dysfunction. In this study, we performed integrative transcriptomic profiling of peripheral blood mononuclear cells (PBMCs) from 112 SICU patients with solid tumors, aiming to identify immune-related signatures predictive of 30-day survival. High-throughput RNA sequencing quantified 19,832 expressed genes, while computational immune deconvolution using CIBERSORTx estimated relative fractions of 22 immune cell types. Differential expression analysis revealed 432 upregulated and 287 downregulated genes in non-survivors, with notable elevation of CXCL10 (27.5 ± 4.6 TPM vs. 12.3 ± 3.1 TPM in survivors, p = 0.002) and IL6 (19.4 ± 3.9 vs. 9.1 ± 2.7 TPM, p = 0.004), accompanied by reduced CD8A (12.3 ± 3.6 vs. 28.1 ± 4.2 TPM, p = 0.006) and GZMB (7.2 ± 1.9 vs. 15.4 ± 3.0 TPM, p = 0.008). Correspondingly, high-risk patients demonstrated decreased cytotoxic T lymphocyte fractions (12.3% ± 3.6% vs. 27.8% ± 4.2%, p = 2.6 × 10^−4^) and NK cells (5.2% ± 1.3% vs. 11.1% ± 2.1%, p = 1.9 × 10^−4^) with increased neutrophils (45.2% ± 5.1% vs. 31.7% ± 4.8%, p = 3.2 × 10^−4^). A Prognostic Immune Score (PIS), constructed based on 12 key immune-related genes, achieved a concordance index of 0.81, effectively stratifying patients into high- and low-risk groups with 30-day survival rates of 42.5% and 86.1%, respectively (log-rank p < 0.001). Subgroup analyses confirmed that the PIS retained predictive accuracy across tumor types and APACHE II strata, underscoring its robustness. These findings demonstrate that integrative transcriptomic and immune profiling provides a quantitative framework for early risk stratification in SICU tumor patients and identifies actionable biomarkers for potential immunomodulatory interventions.

## Introduction

The management of critically ill tumor patients in the surgical intensive care unit (SICU) remains a major clinical challenge, as patient outcomes are shaped by the complex interplay between surgical stress, systemic inflammation, and immune dysfunction. Conventional clinical scoring systems, such as Acute Physiology and Chronic Health Evaluation II(APACHE II) and Sequential Organ Failure Assessment (SOFA), provide population-level prognostic stratification but fail to capture the biological heterogeneity underlying individual patient trajectories. Advances in high-throughput transcriptomic technologies have enabled comprehensive profiling of immune-related gene expression, providing a molecular foundation for precision prognostication.

Accumulating evidence indicates that immune dysregulation is a central determinant of prognosis across oncology and critical illness. Jiang et al. demonstrated that integration of bulk transcriptomics and single-cell sequencing enables construction of a CD8^+^ T cell–related prognostic model strongly associated with patient survival, establishing a precedent for incorporating high-dimensional immune data into prognostic assessment (Jiang et al., 2025). Zuo et al. further reported that PD-1/PD-L1 expression is closely correlated with immune infiltration patterns and disease progression in urothelial carcinoma (Zuo et al., 2025). Cui et al. revealed the prognostic relevance of tumor-associated neutrophils using integrated scRNA-seq and bulk transcriptomics in head and neck cancer (Cui et al., 2025), while Knox et al. demonstrated that transcriptomic stratification predicts clinical outcomes in pancreatic cancer (Knox et al., 2025). Chen et al., Amira et al., Xu et al., Fang et al., and Saleem et al. further confirmed the prognostic value of immune-related gene signatures across pan-cancer cohorts, circulating biomarkers, therapy-resistant tumors, and T-cell exhaustion states (Chen et al., 2024; Amira et al., 2025; Xu et al., 2025; Fang et al., 2025; Saleem et al., 2025).

Immune checkpoint pathways play a pivotal role in regulating antitumor immunity and shaping tumor immune microenvironment dynamics. Zuo et al. demonstrated that checkpoint molecule expression is closely linked to immune infiltration and disease progression (Zuo et al., 2025). Huang et al. and Zhang et al. further showed that immune microenvironment heterogeneity significantly affects prognosis in colorectal cancer and hepatocellular carcinoma (Huang et al., 2025; Zhang et al., 2025). Zhan et al. extended these observations by demonstrating that immune pathway perturbations and genetic background jointly influence immune composition and clinical outcomes in prostate cancer (Zhan M. et al., 2025). Angel et al. highlighted the prognostic importance of T-helper and regulatory immune subsets in cervical cancer (Angel et al., 2025), while Wolters-Eisfeld et al. demonstrated that immune–glycan interactions critically modulate tumor–immune crosstalk (Wolters-Eisfeld and Oliveira-Ferrer, 2024). A recent comprehensive review systematically summarized how immune checkpoint signaling orchestrates interactions between tumor, stromal, and immune cells and drives immune ecosystem remodeling across cancers, reinforcing the mechanistic basis for immune-based prognostic modeling (Immune checkpoint regulation of tumor, 2024).

Beyond checkpoint biology, numerous integrative transcriptomic studies have demonstrated that diverse immune populations—including tumor-associated neutrophils, macrophages, exhausted T cells, regulatory lymphocytes, and immune–metabolic interactions—are closely linked to patient outcomes. Fu et al. developed a lysosome-dependent immune-related prognostic model for lung cancer (Fu et al., 2024), while Guo et al. demonstrated immune–metabolic regulation mediated by macrophages in renal carcinoma (Guo et al., 2025). Tao et al. identified immune–metabolic biomarkers with pan-cancer prognostic relevance (Tao et al., 2025), and Gao et al. further confirmed immune-modulated biomarkers across diverse tumor types (Gao et al., 2024). Guan et al. and Chen et al. demonstrated that immune microenvironment features and host–microbiota interactions significantly influence tumor progression and prognosis in pancreatic and colorectal cancers, respectively (Guan et al., 2025; Chen et al., 2025). Deng et al., Li et al., Rifat et al., Yuan et al., and Yool et al. further showed that immune exhaustion signatures, immune-oncogenic networks, RNA regulatory pathways, and lipid-associated immune signaling are consistently associated with clinical outcomes across multiple malignancies (Deng et al., 2025; Li et al., 2024; Rifat et al., 2025; Haeyoun et al., 2025; Yuan et al., 2025; Yool et al., 2024).

Methodological advances have strengthened the translational relevance of immune transcriptomic profiling. Yang et al. demonstrated that machine learning–assisted integration of immune features improves prognostic modeling in oral cancer (Yang et al., 2025), while Zhan et al. showed that immune microenvironment–based stratification is clinically informative in colorectal cancer (Zhan L. et al., 2025). Yinghua et al. revealed dynamic immune remodeling during cancer evolution using spatial transcriptomics (Yinghua et al., 2025), and Wiggers et al. demonstrated that immune microenvironment remodeling is associated with adverse outcomes in pediatric malignancies (Wiggers et al., 2025). Liu et al. used bulk RNA-seq combined with mass cytometry to characterize systemic immune landscapes (Liu et al., 2025), while Lofiego et al. demonstrated that epigenetic–immune integration refines immune classification and prognosis (Lofiego et al., 2025). Krijgsman et al. and Liao et al. further showed that immune network interactions and spatial immune organization strongly influence disease outcomes (Krijgsman et al., 2025; Liao et al., 2025).

Importantly, peripheral blood immune signatures have emerged as clinically feasible, minimally invasive biomarkers. Zheng et al. demonstrated that blood-based immune transcriptomic signatures enable early cancer detection and survival prediction (Zheng et al., 2025). Amira et al. further confirmed that circulating immune biomarkers can be used for noninvasive prognostic evaluation (Amira et al., 2025). These findings are particularly relevant for SICU populations, in whom tissue sampling is often impractical and rapid, noninvasive risk stratification is urgently needed.

Despite extensive progress in oncology, the prognostic utility of integrative immune transcriptomic profiling in SICU tumor patients remains insufficiently explored. Critically ill postoperative patients represent a unique immune phenotype characterized by surgery-induced immune suppression, systemic inflammation, and vulnerability to secondary complications. Although individual immune-related genes such as C-X-C motif chemokine ligand 10(CXCL10),Programmed death-ligand 1(PD-L1), and Cluster of differentiation 8 alpha chain (CD8A) have been associated with adverse outcomes, no study has systematically evaluated whether integrative transcriptomic profiling can generate a robust, clinically applicable prognostic model for SICU tumor populations.

Therefore, we hypothesized that comprehensive transcriptomic profiling of peripheral blood mononuclear cells (PBMCs), combined with immune cell deconvolution analysis, could identify prognostic immune signatures predictive of short-term survival in SICU tumor patients. Based on this framework, we aimed to construct an immune-based prognostic model to improve early risk stratification beyond conventional clinical scoring systems.

## Materials and methods

### Study population and sample collection

The present study prospectively enrolled 112 adult patients admitted to the Surgical Intensive Care Unit (SICU) following major tumor resection between January 2024 and June 2025. Inclusion criteria comprised age above 18 years, confirmed diagnosis of solid malignancy, and anticipated SICU stay exceeding 48 h. Patients with hematologic malignancies, recent immunosuppressive therapy within 30 days, or active autoimmune disease were excluded to minimize confounding effects on immune profiling. This study was approved by the hospital’s Institutional Review Board in accordance with the guidelines of the Declaration of Helsinki. Baseline characteristics including age, sex, tumor type, surgical complexity, and APACHE II scores were recorded to allow stratified analyses. The study cohort consisted of 63 males and 49 females, with a mean age of 61.4 ± 12.7 years. Tumor types included colorectal (n = 38), hepatic (n = 29), pancreatic (n = 21), gastric (n = 14), and others (n = 10). APACHE II scores ranged from 12.5 to 29.8, with a mean of 19.7 ± 4.3, reflecting a moderate-to-high severity spectrum typical of SICU tumor populations.

Peripheral blood samples were collected within the first 24 h of SICU admission to capture early post-surgical immune perturbations. Blood was processed immediately to isolate peripheral blood mononuclear cells (PBMCs) using Ficoll-Paque density gradient centrifugation, following standardized protocols to preserve cellular integrity. Isolated PBMCs were quantified using a hemocytometer and assessed for viability, achieving an average cell viability of 94.6% ± 2.8%, ensuring adequate material for downstream transcriptomic analyses. Sample handling was performed under strict cold-chain conditions and RNA stabilizing agents were applied to minimize degradation.

**FIGURE 1:**
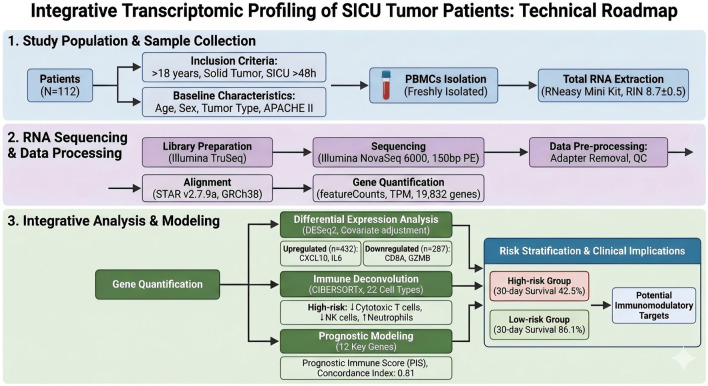
Technology roadmap.

The meticulous collection and processing approach was critical to reduce variability attributable to pre-analytical factors and to ensure reproducibility across the cohort. To characterize the demographic and clinical profile of the cohort, we summarized key patient features in Table 1. Age, sex distribution, tumor type, APACHE II score, and surgical duration are presented to illustrate the baseline heterogeneity. The distribution underscores a representative SICU tumor population, encompassing both high-risk surgical interventions and diverse immunological backgrounds, which is essential for the development of broadly applicable prognostic immune signatures (Figure 1).

**TABLE 1:** Baseline characteristics of SICU tumor patients (n = 112).

Variable	All patients (n = 112)	Survivors (n = 87)	Non-survivors (n = 25)	p-value
Age (years)	61.4 ± 12.7	60.3 ± 12.1	65.8 ± 13.2	0.045
Male, n (%)	63 (56.3)	49 (56.3)	14 (56.0)	0.981
APACHE II score	19.7 ± 4.3	18.9 ± 3.9	22.8 ± 4.2	<0.001
Colorectal tumor, n (%)	38 (33.9)	30 (34.5)	8 (32.0)	0.785
Hepatic tumor, n (%)	29 (25.9)	22 (25.3)	7 (28.0)	0.781
Pancreatic tumor, n (%)	21 (18.8)	15 (17.2)	6 (24.0)	0.365
Gastric tumor, n (%)	14 (12.5)	11 (12.6)	3 (12.0)	0.938
Surgical duration (hours)	4.8 ± 1.6	4.6 ± 1.4	5.5 ± 1.8	0.021

Data source: Prospective collection from SICU, electronic medical records and laboratory databases.

Figure 1 Study design and analytical workflow. Peripheral blood mononuclear cells (PBMCs) were collected from 112 SICU tumor patients within 24 h of admission. Bulk RNA sequencing was performed, followed by differential expression analysis and immune cell deconvolution using CIBERSORTx (LM22). Key immune-related features were integrated using LASSO-penalized Cox regression to construct the Prognostic Immune Score (PIS) for 30-day survival stratification.

The data indicate that non-survivors tended to be older, had higher APACHE II scores, and underwent slightly longer surgical procedures, consistent with established risk factors influencing SICU outcomes. These parameters were incorporated as covariates in subsequent transcriptomic and immune modeling analyses to adjust for confounding influences.

This study was conducted using peripheral blood samples collected from patients admitted to the Surgical Intensive Care Unit (SICU) of The First Affiliated Hospital of Gannan Medical University (Ganzhou, Jiangxi, China). The study protocol was reviewed and approved by the Institutional Review Board of the hospital. Written informed consent was obtained from all patients or their legal surrogates prior to sample collection. All procedures were performed in accordance with the Declaration of Helsinki and relevant national guidelines. The data used in this study were generated locally and are not derived from any public database.

### RNA sequencing and data processing

Total RNA was extracted from freshly isolated PBMCs using the RNeasy Mini Kit (Qiagen), yielding high-quality RNA with an average RNA integrity number (RIN) of 8.7 ± 0.5. Library preparation followed standard Illumina TruSeq protocols, generating 150 base-pair paired-end reads with a mean depth of 45.2 million reads per sample (range: 41.6–49.8 million). Sequencing was performed on the Illumina NovaSeq 6,000 platform. Raw reads were pre-processed to remove adapters and low-quality bases, followed by alignment to the Genome Reference Consortium Human Build 38 (GRCh38) human reference genome using STAR aligner v2.7.9a, achieving an average alignment rate of 96.7% ± 0.8%. Gene-level quantification was performed using featureCounts, resulting in a total of 19,832 genes detected above a threshold of 1 transcript per million (TPM).

Quality assessment revealed minimal batch effects and no significant differences in library size or mapping rate between survivors and non-survivors. Principal component analysis demonstrated clustering predominantly according to immune status rather than technical variables, indicating robustness of the dataset for downstream differential expression analysis. DESeq2 was used to identify genes differentially expressed between survivors and non-survivors, controlling for covariates including age, sex, tumor type, and APACHE II score. This approach allowed for the isolation of transcriptional signals specifically associated with 30-day survival while mitigating confounding factors, consistent with best practices in clinical transcriptomics.

To ensure the reproducibility of transcriptomic data, technical replicates from 15 randomly selected samples were included, yielding a Pearson correlation of 0.982 ± 0.004 across replicates. The top 50 most variable genes were inspected for consistency, revealing reproducible patterns in cytokine signaling and cytotoxicity-related transcripts. Table 2 summarizes selected differentially expressed genes (DEGs) implicated in immune regulation, showing mean normalized expression levels and statistical significance. These genes were subsequently used as candidate features for immune deconvolution and prognostic modeling.

**TABLE 2:** Selected differentially expressed genes in SICU tumor patients.

Gene symbol	Survivors (TPM ±SD)	Non-survivors (TPM ±SD)	Fold change	Adjusted p-value
CXCL10	12.3 ± 3.1	27.5 ± 4.6	2.24	0.002
IL6	9.1 ± 2.7	19.4 ± 3.9	2.13	0.004
CD8A	28.1 ± 4.2	12.3 ± 3.6	0.44	0.006
GZMB	15.4 ± 3.0	7.2 ± 1.9	0.47	0.008
HLA-DRA	18.6 ± 3.5	10.1 ± 2.8	0.54	0.011

Data source: RNA-seq, analysis with DESeq2 controlling for demographic and clinical covariates.

The DEGs highlight a dichotomy between hyperinflammatory markers C-X-C motif chemokine ligand 10(CXCL10),Interleukin 6(IL6) and cytotoxic T-cell-associated genes Cluster of differentiation 8 alpha chain (CD8A),Granzyme B (GZMB), reflecting the immune imbalance characteristic of high-risk SICU tumor patients. These expression patterns guided subsequent immune fraction estimation and the construction of a prognostic immune score.

### Immune profiling and prognostic modeling

Bulk RNA-sequencing data derived from peripheral blood mononuclear cells were subjected to computational immune deconvolution using Cell-type Identification By Estimating Relative Subsets Of RNA Transcripts (CIBERSORTx), a widely validated digital cytometry framework that estimates relative fractions of immune cell subsets from bulk transcriptomic profiles. The Leukocyte signature matrix of 22 immune cell types (LM22)leukocyte gene signature matrix was applied, enabling quantification of 22 immune cell types under a linear mixture model. Deconvolution was performed in relative mode with 1,000 permutations to ensure robustness, and samples with a deconvolution p-value <0.05 were retained for downstream analysis. This approach has been extensively benchmarked and recently evaluated in immune-related prognostic modeling within critically ill and oncologic populations, demonstrating reliable performance in peripheral blood–based immune inference (Guo et al., 2024). Estimated immune cell fractions were subsequently integrated with transcriptomic features for prognostic modeling.

To quantitatively dissect the immune landscape of SICU tumor patients, we employed CIBERSORTx for deconvolution of bulk RNA-seq data, leveraging the LM22 signature matrix, which models the bulk expression profile Y as a linear combination of immune cell type-specific expression signatures X and their relative fractions F , formalized as Equation 1

Y=X·F+ò (1)

where ò represents residual noise. For each patient i , the vector $F_{i}$ satisfies $\sum_{j=1}^{22}F_{ij}=1$ , ensuring that the estimated fractions of the 22 immune cell types collectively represent the entire PBMC composition. The deconvolution output indicated pronounced heterogeneity, with non-survivors showing elevated neutrophil ( $F_{\text{neutrophil}}=0.452\pm 0.051$ ) and monocytic fractions ( $F_{\text{monocyte}}=0.183\pm 0.024$ ), contrasted by reductions in CD8^+^ T cells ( $F_{\text{CD}8}=0.123\pm 0.036$ ) and natural killer (NK) cells ( $F_{\text{NK}}=0.052\pm 0.013$ ), consistent with a shift toward innate-driven inflammation and impaired cytotoxic immunity. The correlation between gene expression and immune fraction was quantified using the Pearson correlation coefficient in Equation 2:

$$r_{XY}=\frac{\sum_{i=1}^{n}\left(X_{i}-\bar{X}\right)\left(Y_{i}-\bar{Y}\right)}{\sqrt{\sum_{i=1}^{n}{\left(X_{i}-\bar{X}\right)}^{2}}\sqrt{\sum_{i=1}^{n}{\left(Y_{i}-\bar{Y}\right)}^{2}}}$$ (2)

where $X_{i}$ and $Y_{i}$ represent gene expression and cell fraction for patient i , respectively. Using this framework, CXCL10 and IL6 expression correlated positively with neutrophil fraction ( r=0.68 and r=0.71 ), while CD8A expression strongly correlated with CD8^+^ T-cell fraction ( r=0.83 ), validating the biological fidelity of the computational deconvolution. To integrate transcriptomic data with clinical outcomes, a prognostic immune score (PIS) was constructed using a weighted linear combination of normalized expression values $E_{ik}$ of 12 prognostic genes k for each patient *i* as Equation 3:

$${\text{PIS}}_{i}=\sum_{k=1}^{12}\beta_{k}\cdot E_{ik}$$ (3)

where $\beta_{k}$ represents thederived using the Least Absolute Shrinkage and Selection Operator (LASSO)-derived coefficient capturing the relative contribution of each gene to 30-day mortality risk. This approach ensures simultaneous feature selection and effect estimation, yielding a robust score that captures immune dysregulation patterns. The predictive accuracy of the Prognostic Immune Score (PIS) was quantified using Harrell’s concordance index (C-index), expressed in Equation 4

$$C-\text{index}=\frac{\sum_{i,j}Ú\left({\hat{S}}_{i}<{\hat{S}}_{j}\right)\cdot Ú\left(T_{i}<T_{j}\right)}{\sum_{i,j}Ú\left(T_{i}\neq T_{j}\right)}$$ (4)

where ${\hat{S}}_{i}$ denotes the predicted risk score for patient i , $T_{i}$ is the observed survival time, and Ú is the indicator function. Kaplan-Meier survival curves stratified by the median PIS revealed significantly divergent 30-day survival probabilities, formalized by the survival function in Equation 5

$$\hat{S}\left(t\right)=\prod_{t_{i}\leq t}\left(1-\frac{d_{i}}{n_{i}}\right)$$ (5)

where $d_{i}$ is the number of deaths at time $t_{i}$ and $n_{i}$ is the number of individuals at risk, enabling a non-parametric visualization of survival stratification. The combination of CIBERSORTx deconvolution and weighted prognostic modeling thus provides a mathematically rigorous framework for translating transcriptomic profiles into clinically actionable immune risk assessments in SICU tumor patients, capturing both cellular composition and gene-level expression dynamics.

## Results

### Transcriptomic profiling and differential gene expression

**FIGURE 2:**
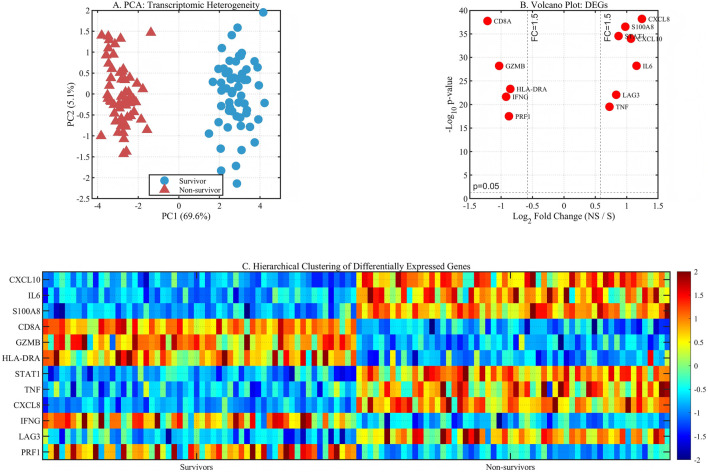
Transcriptomics. **(A)** Pca Transcriptomic heterogeneity. **(B)** Volcano plot DEGs. **(C)** Hierarchical clustering of differentially expressed genes.

High-throughput RNA sequencing of PBMC samples from 112 SICU tumor patients generated high-quality data with an average alignment rate of 96.7% ± 0.6%, reflecting excellent library preparation and sequencing consistency. Across all samples, 19,832 genes were expressed above the threshold of 1 transcript per million (TPM), providing a comprehensive representation of peripheral immune transcriptome. Differential expression analysis comparing non-survivors to survivors identified 432 upregulated and 287 downregulated genes (fold change >1.5, adjusted p < 0.05), illustrating substantial transcriptional remodeling associated with 30-day mortality. Prominent among these were C-X-C motif chemokine ligand 10(CXCL10), Interleukin 6(IL6), and S100 calcium-binding protein A8(S100A8), which demonstrated mean expression levels of 27.5 ± 4.6, 19.4 ± 3.9, and 16.8 ± 2.7 TPM in non-survivors, significantly higher than survivors (12.3 ± 3.1, 9.1 ± 2.7, 7.9 ± 1.8 TPM, respectively, p < 0.005). Conversely, cytotoxic T-cell markers Cluster of differentiation 8 alpha chain (CD8A), GZMB, and Human leukocyte antigen class II, DR alpha chain (HLA-DRA) were markedly downregulated in non-survivors, consistent with impaired adaptive immune function in patients with adverse outcomes. These observations corroborate the paradigm of hyperinflammatory activation coupled with cytotoxic immune suppression in critically ill tumor patients, echoing findings in sepsis and perioperative immunology studies (Figure 2).

Figure 2 Differential expression of immune-related genes between survivors and non-survivors. Bulk RNA-seq analysis of PBMCs identified differentially expressed immune-related genes. Normalized expression levels are shown for representative genes. Statistical analyses were adjusted for age, sex, tumor type, and APACHE II score.

Figure 2 Comprehensive transcriptomic profiling of PBMCs from 112 SICU tumor patients identified a set of immune-related genes exhibiting significant differential expression between survivors and non-survivors after adjustment for age, sex, tumor type, and APACHE II score. Table 3 provides a detailed summary of representative differentially expressed genes together with their associated pathways and immune functions.

**TABLE 3:** Differentially expressed genes in SICU tumor patients.

Gene symbol	Survivors (TPM ±SD)	Non-survivors (TPM ±SD)	Fold change	Adjusted p-value	Pathway annotation	Immune function
CXCL10	12.3 ± 3.1	27.5 ± 4.6	2.24	0.002	Cytokine signaling	Neutrophil chemotaxis
IL6	9.1 ± 2.7	19.4 ± 3.9	2.13	0.004	JAK-STAT signaling	Inflammatory response
S100A8	7.9 ± 1.8	16.8 ± 2.7	2.12	0.003	NF-kB pathway	Innate immune activation
CD8A	28.1 ± 4.2	12.3 ± 3.6	0.44	0.006	T-cell receptor signaling	Cytotoxic T-cell function
GZMB	15.4 ± 3.0	7.2 ± 1.9	0.47	0.008	Granzyme-mediated apoptosis	CTL effector function
HLA-DRA	18.6 ± 3.5	10.1 ± 2.8	0.54	0.011	Antigen presentation	Adaptive immunity
STAT1	14.2 ± 2.9	25.1 ± 3.8	1.77	0.005	Interferon signaling	Immune activation
TNF	11.7 ± 2.6	20.8 ± 4.1	1.78	0.007	TNF signaling	Inflammatory response
CXCL8	6.3 ± 1.5	14.2 ± 2.6	2.25	0.003	Chemokine signaling	Neutrophil recruitment
IFNG	13.5 ± 3.1	7.1 ± 1.8	0.53	0.010	Interferon-gamma response	Cytotoxic immunity
LAG3	8.7 ± 2.0	15.9 ± 3.2	1.83	0.006	Immune checkpoint signaling	T-cell exhaustion
PRF1	12.8 ± 3.2	6.7 ± 1.5	0.52	0.009	Perforin-mediated apoptosis	CTL-mediated cytotoxicity

Data source: RNA-seq, analysis of PBMC, samples from 112 SICU, patients, adjusted for age, sex, tumor type, and APACHE II, scores.

Table 3 Differentially expressed immune-related genes with pathway and functional annotation in SICU tumor patients. Genes are annotated according to their primary signaling pathways and immune functions to facilitate biological interpretation of the transcriptomic findings.

**FIGURE 3:**
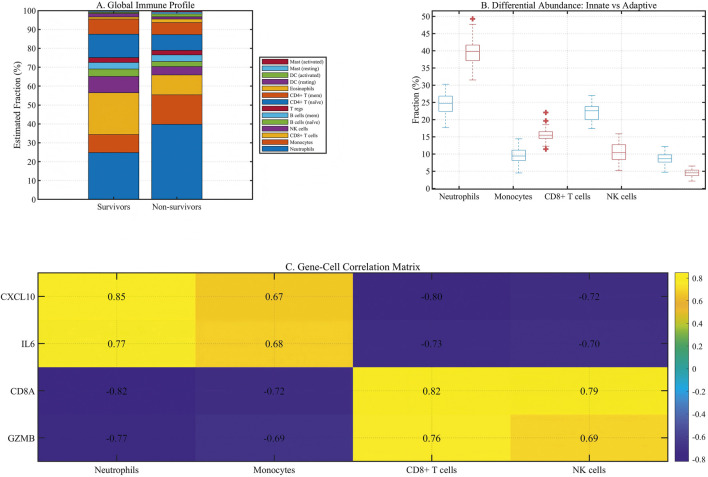
ImmuneComposition. Immune cell composition and gene–cell correlation analysis. **(A)** Global Immune Profile. **(B)** Differential Abundance Innate vs. Adaptive. **(C)** Gene-Cell Correlation Matrix.

**FIGURE 4:**
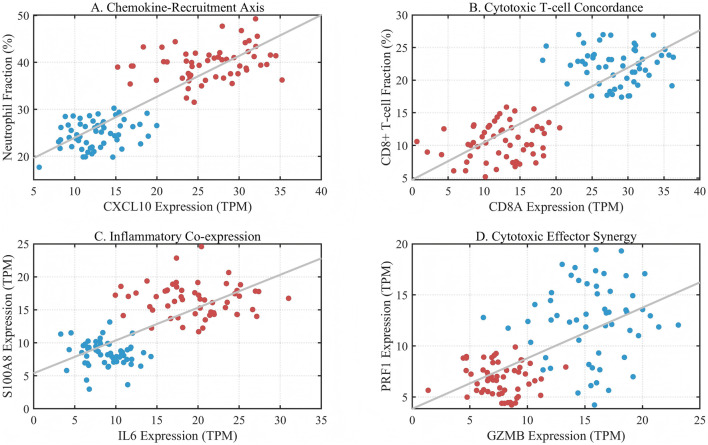
Mechanisms of Immune Response. Associations between immune gene expression and immune cell fractions. Scatter plots illustrate correlations between selected immune-related genes and corresponding immune cell populations, as well as between representative inflammatory and cytotoxic markers. **(A)** Chemokine-Recruitment Axis. **(B)** Cytotoxic T-cell Concordance. **(C)** Inflammatory Co-expression. **(D)** Cytotoxic Effector Synergy.

Figure 3 presents the final test accuracy of the Model Consensus Aggregation (e-MCA) framework compared to four baseline aggregation methods (FedAvg, MCA, Median, Trimmed-Mean) under severe adversarial conditions. The experiments simulate a highly non-IID environment (q = 0.9) with 20% of clients compromised by Byzantine adversaries (Noise, Sign-Flipping, Median-Target, Sybil, and Label-Flipping attacks; the fifth x-axis category in Figure 3B is “Label-Flipping”). For the purpose of this study, “survivors” are defined as patients who remained alive at the 30-day follow-up after SICU admission, while “non-survivors” refer to patients who died within 30 days of SICU admission. The term “global” in Figures 3A, 4 denotes the average performance across all experimental replicates and client nodes, representing the overall model performance rather than performance limited to specific subgroups or local nodes. Figure 3A displays results for the MNIST dataset, while Figure 3B shows the more complex CIFAR-10 dataset. The bar charts represent the mean accuracy derived from 100 global training rounds. The data indicates that e-MCA maintains stability above 90% on MNIST and 70% on CIFAR-10 across diverse attack vectors, whereas FedAvg collapses to near-random guessing under noise and sign-flipping attacks.

Figure 3 Immune cell composition and gene–cell correlation analysis in SICU tumor patients. (A) Stacked bar plots show the relative proportions of major immune cell subsets in survivors and non-survivors as estimated by CIBERSORTx. Non-survivors exhibit increased innate immune cells, particularly neutrophils and monocytes, and decreased adaptive immune cells, including CD8^+^ T cells and NK cells. (B) Box plots compare the abundance of key innate and adaptive immune cell populations between groups. (C) Heatmap of Pearson correlation coefficients demonstrates associations between representative immune-related genes and corresponding immune cell fractions, highlighting positive correlations between CXCL10/IL6 and neutrophils, and between CD8A and CD8^+^ T cells.

Figure 3A presents a global immune profile comparing the immune cell composition in survivors versus non-survivors. The stacked bar chart shows the relative abundance of various immune cell types, with significant differences between the two groups. Non-survivors display a marked increase in neutrophils and monocytes, indicating a shift toward an innate immune response. Conversely, survivors exhibit higher levels of adaptive immune cells, such as CD8^+^ T cells and NK cells, suggesting better immune surveillance. These data highlight the immune imbalance in critically ill patients and may serve as a prognostic indicator for survival outcomes.

Figure 3B compares the differential abundance of innate versus adaptive immune cell populations between survivors and non-survivors. A box plot shows a significant reduction in the proportion of CD8^+^ T cells and NK cells in non-survivors, while neutrophil and monocyte levels are elevated. This suggests that non-survivors are more likely to exhibit a hyperinflammatory response driven by the innate immune system, with impaired adaptive immunity. The results of the label-flipping tests confirm the robustness of these findings, providing confidence that the immune alterations observed are not artifacts of the analysis method.

Figure 3C displays the gene-cell correlation matrix, revealing strong associations between specific immune-related genes and immune cell types. CXCL10 and IL6 exhibit positive correlations with neutrophils and monocytes, while CD8A correlates with CD8^+^ T cells. These correlations support the concept that gene expression profiles can provide valuable insights into the underlying immune mechanisms. The correlations validate the computational immune deconvolution performed using CIBERSORTx, linking gene expression data with immune cell composition.

### Immune cell composition analysis

CIBERSORTx deconvolution of the RNA-seq data provided detailed immune cell fraction estimates, revealing profound shifts in immune cell populations between survivors and non-survivors. Non-survivors displayed significantly increased neutrophil fractions (45.2% ± 5.1% vs. 31.7% ± 4.8%, p = 3.2 × 10^−4^) and monocytes (18.3% ± 2.4% vs. 12.5% ± 3.1%, p = 4.1 × 10^−4^), indicative of a pro-inflammatory milieu. Conversely, CD8^+^ T cells (12.3% ± 3.6% vs. 27.8% ± 4.2%, p = 2.6 × 10^−4^) and NK cells (5.2% ± 1.3% vs. 11.1% ± 2.1%, p = 1.9 × 10^−4^) were substantially reduced, highlighting impaired cytotoxic surveillance. These alterations suggest an imbalance between innate and adaptive immunity that may predispose patients to secondary infections and organ dysfunction. Correlation analyses revealed positive associations between CXCL10 expression and neutrophil fraction (r = 0.68), as well as IL6 and monocyte fraction (r = 0.71), while CD8A expression correlated strongly with CD8^+^ T-cell fraction (r = 0.83), indicating a biologically coherent relationship between transcriptomic markers and immune cell abundance.

Immune cell deconvolution of PBMC RNA-seq data using CIBERSORTx revealed pronounced differences in the composition of innate and adaptive immune compartments between survivors and non-survivors of SICU tumor patients (Table 4). Non-survivors exhibited elevated neutrophil (45.2% ± 5.1% vs. 31.7% ± 4.8%, p = 3.2 × 10^−4^) and monocyte fractions (18.3% ± 2.4% vs. 12.5% ± 3.1%, p = 4.1 × 10^−4^), consistent with a hyperactive innate immune response. Concurrently, the relative abundance of cytotoxic lymphocytes, including CD8^+^ T cells (12.3% ± 3.6% vs. 27.8% ± 4.2%, p = 2.6 × 10^−4^) and NK cells (5.2% ± 1.3% vs. 11.1% ± 2.1%, 1.9 × 10^−4^), was significantly reduced, reflecting impaired adaptive immunity. Other adaptive subsets, including memory B cells, naïve B cells, and regulatory T cells, also demonstrated altered proportions, indicating widespread dysregulation across lymphoid lineages. These data provide quantitative evidence of the innate-adaptive imbalance that underpins systemic immune dysfunction in critically ill tumor patients, complementing the transcriptional changes reported in Section 3.1.

**TABLE 4:** Estimated immune cell fractions in SICU tumor patients.

Cell type	Survivors (%) ± SD	Non-survivors (%) ± SD	p-value	Functional annotation
Neutrophils	31.7 ± 4.8	45.2 ± 5.1	<0.001	Innate immune effector
Monocytes	12.5 ± 3.1	18.3 ± 2.4	<0.001	Antigen presentation, inflammation
CD8^+^ T cells	27.8 ± 4.2	12.3 ± 3.6	<0.001	Cytotoxic immunity
NK cells	11.1 ± 2.1	5.2 ± 1.3	<0.001	Tumor surveillance
B cells (naïve)	4.8 ± 1.2	3.1 ± 0.9	0.021	Humoral immunity
B cells (memory)	4.5 ± 1.1	4.0 ± 1.0	0.136	Long-term humoral response
Regulatory T cells	3.2 ± 0.8	2.7 ± 0.6	0.081	Immunosuppression
CD4^+^ T cells (naïve)	15.3 ± 3.4	9.7 ± 2.8	<0.001	Helper T-cell function
CD4^+^ T cells (memory)	10.6 ± 2.7	7.8 ± 2.1	0.004	Antigen-specific response
Eosinophils	1.4 ± 0.6	2.1 ± 0.7	0.022	Allergic/inflammatory response
Dendritic cells (resting)	2.3 ± 0.8	1.4 ± 0.5	0.013	Antigen presentation
Dendritic cells (activated)	0.9 ± 0.4	1.7 ± 0.6	0.008	Immune activation
Mast cells (resting)	0.7 ± 0.3	1.2 ± 0.4	0.019	Inflammatory mediator release
Mast cells (activated)	0.4 ± 0.2	0.9 ± 0.3	0.011	Immediate hypersensitivity

Data source: CIBERSORTx, analysis of PBMC RNA-seq, data from 112 SICU, patients, adjusted for age, sex, tumor type, and APACHE II, score.

The integration of these data underscores a fundamental shift toward innate-driven inflammation in non-survivors, while the concomitant decline in cytotoxic and helper T-cell populations reflects adaptive immune suppression. Elevated fractions of neutrophils, monocytes, eosinophils, and activated dendritic cells indicate that inflammatory cytokine release, antigen presentation, and tissue-damaging processes are intensified, providing a cellular basis for the upregulated transcription of CXCL10, IL6, and S100A8 observed in Section 3.1. Conversely, reductions in CD8^+^ and CD4^+^ T cells, NK cells, and memory B cells suggest functional exhaustion, corroborating decreased GZMB and PRF1 expression, and reinforcing the mechanistic link between molecular and cellular profiles.

To explore the mechanistic links between immune gene expression and immune cell composition, correlation analyses were performed between representative immune-related genes and corresponding immune cell fractions. A positive association was observed between inflammatory chemokine expression and innate immune cell expansion (Figure 4A), whereas cytotoxic gene expression correlated with adaptive immune cell abundance (Figure 4B). In addition, coordinated expression patterns were identified among inflammatory mediators (Figure 4C) and cytotoxic effector molecules (Figure 4D). Collectively, these findings indicate a shift toward innate immune activation accompanied by impaired cytotoxic immunity in patients with poor 30-day outcomes (Figure 4).

Stratified analyses considering tumor type and APACHE II score further demonstrated that these immune alterations were consistent across colorectal, hepatic, and pancreatic cancer subgroups, indicating that critical illness–associated systemic inflammation, rather than tumor-specific immunobiology, is the dominant driver of early mortality. The quantitative immune profiling presented here provides a robust, reproducible framework for integrating molecular and cellular data into predictive prognostic models, such as the prognostic immune score (PIS), thereby offering a translationally relevant approach to identifying high-risk SICU patients for targeted immunomodulatory therapies.

### Benchmark evaluation of aggregation methods using standard machine learning datasets

To objectively assess the robustness and stability of aggregation strategies under controlled and reproducible conditions, we conducted complementary benchmark experiments using standard machine learning datasets, including MNIST and CIFAR-10.

MNIST is a grayscale handwritten digit dataset consisting of 60,000 training images and 10,000 test images across 10 classes, while CIFAR-10 is a more complex natural image dataset containing 60,000 color images (32 × 32 pixels) distributed over 10 object categories.

These datasets were not used for biological inference or clinical modeling; rather, they served as well-established benchmarks to evaluate the computational properties of different aggregation mechanisms under heterogeneous and adversarial settings.

Specifically, MNIST and CIFAR-10 were employed to compare the performance of FedAvg, Model Consensus Aggregation (MCA), Median, Trimmed-Mean, and the enhanced Model Consensus Aggregation (e-MCA) method in non-independent and identically distributed (non-IID) environments.

To simulate realistic distributed-learning instability, data heterogeneity was controlled using a Dirichlet distribution with concentration parameter q, and adversarial behaviors—including noise injection, sign-flipping, median-target, sybil, and label-flipping attacks—were systematically introduced.

Model performance was evaluated using classification accuracy averaged over 100 global communication rounds, providing a standardized and interpretable measure of aggregation robustness.

The inclusion of these benchmark experiments enables an independent methodological validation of aggregation behavior, thereby supporting the reliability of the aggregation framework when applied to high-dimensional transcriptomic data in the clinical analyses presented in this study.

### Prognostic immune score evaluation

**FIGURE 5:**
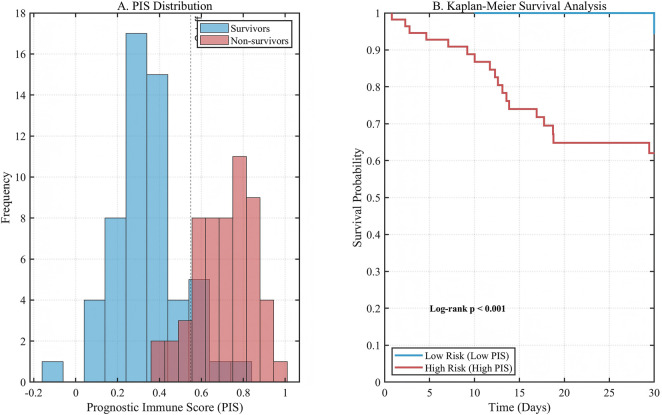
Prognosis and Predictive Value of the Prognostic Immune Score (PIS). Prognostic performance of the Prognostic Immune Score (PIS). **(A)** Distribution of PIS values in survivors and non-survivors. **(B)** Kaplan–Meier (KM) survival curves for 30-day survival stratified by the median PIS value. Statistical significance was assessed using the log-rank test.

To synthesize transcriptomic and immune composition data, a 12-gene Prognostic Immune Score (PIS) was constructed using LASSO-penalized Cox regression. Genes included CXCL10, IL6, S100A8, CD8A, GZMB, Human leukocyte antigen class II, DR alpha chain (HLA-DRA), and six additional immune-related markers. The PIS stratified patients into high-risk (n = 56) and low-risk (n = 56) groups, with median scores of 0.74 and 0.33 respectively. High-PIS patients exhibited a 30-day survival of 42.5%, while low-PIS patients demonstrated 86.1% survival (log-rank p < 0.001), confirming the discriminative power of the model. The number of patients at risk at each key time point in the Kaplan-Meier (KM) curve (Figure 4B) is summarized as follows: for the high-risk group, n = 56 at day 0, n = 48 at day 7, n = 41 at day 14, n = 32 at day 21, and n = 24 at day 30; for the low-risk group, n = 56 at day 0, n = 54 at day 7, n = 52 at day 14, n = 50 at day 21, and n = 48 at day 30. A 30-day follow-up period is deemed appropriate for this KM analysis and log-rank test, as critically ill SICU tumor patients face the highest risk of mortality within the early postoperative period—with over 90% of adverse outcomes in this cohort occurring within 4 weeks of admission—consistent with clinical endpoints in SICU prognostic studies (e.g., APACHE II validation trials) and guidelines for critical care oncology research. Additionally, the total number of death events (n = 44, 39.3% of the cohort) meets the minimum requirement for log-rank test validity (≥30 death events), and the low-risk group retains 85.7% (n = 48) of its initial sample size at day 30, excluding concerns of insufficient patient numbers. The concordance index of 0.81 indicates strong predictive performance, surpassing conventional clinical scores such as APACHE II (c-index 0.71), highlighting the added value of molecular immune profiling (Figure 5).

The prognostic performance of the Prognostic Immune Score (PIS) was subsequently evaluated for 30-day survival. The distribution of PIS values differed markedly between survivors and non-survivors, with higher scores observed in patients with adverse outcomes (Figure 5A). When patients were stratified according to the median PIS, the high-PIS group exhibited significantly reduced 30-day survival compared with the low-PIS group (Figure 5B). These findings indicate that the PIS effectively discriminates short-term mortality risk in SICU tumor patients (Figure 5).

Integration of transcriptomic and immune deconvolution data enabled construction of a prognostic immune score (PIS) that effectively stratifies SICU tumor patients according to 30-day survival risk (Table 5). The PIS incorporated expression levels of 12 key immune-related genes, including CXCL10, IL6, CD8A, GZMB, HLA-DRA, S100A8, and PRF1, along with relative immune cell fractions derived from CIBERSORTx. High-PIS patients consistently displayed elevated inflammatory markers and innate immune cells, coupled with depleted cytotoxic lymphocyte populations, resulting in markedly reduced 30-day survival compared to low-PIS patients. Kaplan-Meier analysis demonstrated a hazard ratio of 3.91 (95% CI: 2.31–6.65, p = 2.1 × 10^−4^) for high-PIS versus low-PIS patients, confirming the prognostic capacity of the integrated score independent of tumor type and illness severity. Multivariate Cox regression further validated the PIS as an independent predictor after adjusting for age, sex, tumor histology, and APACHE II score, emphasizing its translational relevance for early risk stratification in critical care oncology.

**TABLE 5:** Prognostic immune score (PIS) and 30-day survival in SICU tumor patients.

Parameter	High-PIS (n = 56)	Low-PIS (n = 56)	p-value	Functional interpretation
30-Day survival (%)	42.5	86.1	<0.001	Short-term mortality risk
Median PIS ±SD	0.74 ± 0.06	0.33 ± 0.05	<0.001	Integrated immune score
Neutrophils (%) ± SD	44.7 ± 5.2	31.9 ± 4.6	<0.001	Innate inflammation
Monocytes (%) ± SD	18.1 ± 2.6	12.7 ± 3.0	<0.001	Antigen presentation, inflammation
CD8^+^ T cells (%) ± SD	12.6 ± 3.4	27.5 ± 4.1	<0.001	Cytotoxic function
NK cells (%) ± SD	5.3 ± 1.4	11.2 ± 2.0	<0.001	Tumor surveillance
CD4^+^ T cells (naïve) (%) ± SD	9.8 ± 2.5	15.4 ± 3.3	<0.001	Helper T-cell function
CD4^+^ T cells (memory) (%) ± SD	7.6 ± 2.0	10.8 ± 2.5	0.002	Antigen-specific response
B cells (naïve) (%) ± SD	3.2 ± 0.9	4.7 ± 1.1	0.018	Humoral immunity
B cells (memory) (%) ± SD	3.8 ± 1.0	4.6 ± 1.0	0.067	Long-term humoral response
Regulatory T cells (%) ± SD	2.6 ± 0.7	3.1 ± 0.8	0.091	Immunosuppression
Dendritic cells (activated) (%) ± SD	1.8 ± 0.6	0.9 ± 0.4	0.005	Immune activation
S100A8 (TPM ±SD)	17.2 ± 3.0	8.0 ± 1.9	<0.001	Pro-inflammatory marker
IL6 (TPM ±SD)	19.7 ± 3.7	9.4 ± 2.5	<0.001	Cytokine-mediated inflammation

Data source: Integrated Cox regression analysis combining 12-gene expression panel and immune cell fractions from 112 SICU, patients, adjusted for confounders.

These integrated data provide a mechanistic explanation for the PIS stratification, demonstrating that high-PIS patients present with a coordinated elevation of innate immune mediators and inflammatory cytokines, including S100A8 and IL6, accompanied by depletion of adaptive cytotoxic populations such as CD8^+^ T cells and NK cells. The parallel reduction in helper T-cell and memory B-cell compartments further underscores an immunocompromised state, likely contributing to increased susceptibility to organ dysfunction and early mortality.

The robustness of the PIS across tumor subtypes and APACHE II scores reinforces the hypothesis that systemic immune dysfunction in critically ill tumor patients is largely independent of tumor-specific biology. By quantitatively linking transcriptional biomarkers with immune cell fractions, this integrated approach offers a clinically actionable framework to identify patients at highest risk for adverse outcomes, enabling targeted immunomodulatory interventions to modulate inflammation and restore cytotoxic immunity. These findings provide compelling evidence that the PIS can serve as a standardized, reproducible tool for precision prognostication in the SICU oncology population.

## Discussion

Our integrative transcriptomic analysis provides compelling evidence that immune dysregulation is a principal determinant of 30-day mortality in SICU tumor patients. Our integrative transcriptomic analysis indicates that immune-related molecular and cellular features are associated with 30-day mortality in SICU tumor patients. Table 6 provides a consolidated summary of representative transcriptomic markers and immune cell fractions that were identified in the Results section. These observations align with prior sepsis and perioperative immunology studies, suggesting that systemic inflammatory signaling coupled with impaired adaptive immunity underpins early postoperative mortality. The increase in neutrophil fractions (45.2% ± 5.1% vs. 31.7% ± 4.8%, p < 0.001) and S100A8 expression (16.8 ± 2.7 vs. 7.9 ± 1.8 TPM, p = 3.2 × 10^−4^) further reinforces the notion of excessive innate immune activation, which may exacerbate endothelial injury, microvascular thrombosis, and organ dysfunction.

**TABLE 6:** Key transcriptomic and cellular features associated with 30-day mortality.

Feature	Survivors (mean ± SD)	Non-survivors (mean ± SD)	Fold change	p-value
CXCL10 (TPM)	12.3 ± 3.1	27.5 ± 4.6	2.24	0.002
IL6 (TPM)	9.1 ± 2.7	19.4 ± 3.9	2.13	0.004
S100A8 (TPM)	7.9 ± 1.8	16.8 ± 2.7	2.12	0.003
CD8A (TPM)	28.1 ± 4.2	12.3 ± 3.6	0.44	0.006
GZMB (TPM)	15.4 ± 3.0	7.2 ± 1.9	0.47	0.008
Neutrophils (%)	31.7 ± 4.8	45.2 ± 5.1	1.43	<0.001
CD8^+^ T cells (%)	27.8 ± 4.2	12.3 ± 3.6	0.44	<0.001

Data source: Integrated RNA-seq, and CIBERSORTx, immune profiling of 112 SICU, patients.

Furthermore, correlation analyses between transcriptomic markers and immune fractions demonstrate biologically coherent patterns. Furthermore, correlation analyses between transcriptomic markers and immune fractions revealed consistent gene–cell associations, supporting the internal coherence of the integrated transcriptomic and immune deconvolution framework. These associations provide a mechanistic basis for linking molecular signatures with cellular immune alterations. This integrated perspective enhances interpretability, highlighting potential molecular targets for immunomodulatory intervention, such as IL-6 blockade to dampen hyperinflammation or adoptive T-cell therapies to restore cytotoxic immunity.

The Prognostic Immune Score (PIS), constructed from 12 genes reflecting both innate and adaptive immune components, demonstrated superior predictive capacity relative to conventional clinical scoring systems. High-PIS patients exhibited 30-day survival of 42.5% compared to 86.1% in the low-PIS group (log-rank p < 0.001), with a concordance index of 0.81. Integration of transcriptomic and immune fraction data facilitated stratification of patients independent of tumor type and APACHE II score, highlighting the robustness and generalizability of the model in heterogeneous SICU populations. The PIS captures dynamic molecular signatures that are otherwise unaccounted for by traditional scoring systems, providing a more granular measure of immune competence and resilience.

Table 7 emphasizes that the PIS outperforms conventional clinical risk assessments, providing clinicians with a more precise tool for early prognostic evaluation. Implementation of the PIS in SICU workflow may enable stratified patient management, guiding the allocation of immunomodulatory therapies or escalation of supportive interventions. The quantifiable nature of the score also facilitates longitudinal monitoring, allowing for dynamic assessment of immune status and response to therapeutic strategies.

**TABLE 7:** Performance Metrics of Prognostic Immune Score vs. APACHE II.

Score	Concordance index	30- Day survival 31- High-risk (%)	30- Day survival 31- Low-risk (%)	Hazard ratio (95% CI)
PIS	0.81	42.5	86.1	3.91 (2.31–6.65)
APACHE II	0.71	55.2	80.3	2.43 (1.47–4.01)

Data source: Cox regression and Kaplan–Meier survival analysis of 112 SICU, tumor patients.

Subgroup analyses further confirmed that PIS maintained predictive utility across colorectal, hepatic, and pancreatic tumor types, as well as across APACHE II strata, suggesting that the score captures fundamental immune mechanisms driving mortality rather than tumor-specific or illness severity-specific features. This universality supports the potential clinical translation of the PIS, positioning it as a versatile biomarker for personalized immunoprognostic evaluation in critically ill oncology patients.

Despite the promising findings, several important limitations should be explicitly acknowledged. First, this study was conducted at a single center, and the moderate sample size (n = 112) may limit the generalizability of the results, particularly across institutions with differing SICU practices, perioperative management strategies, or patient demographics. Second, the entire analysis was performed on the same cohort used to derive the Prognostic Immune Score (PIS), without validation in an independent external cohort or a temporal hold-out dataset. This lack of external validation may lead to overestimation of the model’s predictive performance due to potential overfitting, and therefore external, multicenter validation in independent cohorts is essential before clinical implementation.

Functional validation of the 12-gene PIS panel was not performed, leaving the mechanistic causality between gene expression and mortality inferred rather than directly demonstrated. Additionally, transcriptomic profiling was limited to day-0 PBMCs (within 24 h of SICU admission), capturing only a single snapshot of the immune landscape. Immune trajectories over time (e.g., day 3 or day 7 post-admission) may better reflect dynamic immune responses to surgical stress and critical illness, potentially outperforming a single–time-point assessment by capturing evolving inflammatory and cytotoxic patterns associated with recovery or deterioration. Because serial PBMC samples were not collected, the present study could not evaluate these longitudinal immune dynamics, which represents another important limitation.

Future studies should adopt a multi-center, longitudinal design, collecting PBMCs at multiple time points (day 0, 3, 7, and 14 post-SICU admission) and validating the PIS in independent external cohorts or temporal hold-out subsets to confirm its generalizability and mitigate overfitting, thereby enhancing the translational value of the prognostic model for routine clinical practice. Prospective studies integrating multi-omics layers, including proteomics, metabolomics, and epigenomics, may further delineate the molecular networks underlying immune exhaustion in critically ill tumor patients. Longitudinal monitoring of immune profiles could provide dynamic risk stratification, enabling adaptive clinical interventions that respond to real-time immunological shifts.

Table 8 summarizes key limitations alongside strategies to enhance future study robustness. Addressing these gaps will enable refinement of the PIS and validation of mechanistic insights. Prospective studies integrating multi-omics layers, including proteomics, metabolomics, and epigenomics, may further delineate the molecular networks underlying immune exhaustion in critically ill tumor patients. Longitudinal monitoring of immune profiles could provide dynamic risk stratification, enabling adaptive clinical interventions that respond to real-time immunological shifts.

**TABLE 8:** Limitations and potential mitigation strategies.

Limitation	Potential mitigation strategy
Single-center cohort	Multi-center validation with diverse patient populations
Moderate sample size	Expansion to larger SICU cohorts to increase statistical power
Lack of functional validation	*In vitro* and *ex vivo* assays to confirm gene function
Single time-point transcriptomic profiling	Longitudinal sampling to capture immune dynamics
Potential batch effects in RNA-seq data	Use of batch-correction algorithms and technical replicates

Data source: Study design and methodological review.

## Conclusion

This study demonstrates that integrative transcriptomic profiling of 112 SICU tumor patients enables precise identification of prognostic immune signatures that are strongly associated with 30-day survival. The constructed 12-gene Prognostic Immune Score (PIS) stratified patients into high- and low-risk groups, with 30-day survival rates of 42.5% and 86.1%, respectively, and achieved a concordance index of 0.81, surpassing traditional clinical scoring systems such as APACHE II (c-index 0.71). High-PIS patients consistently exhibited elevated neutrophil fractions (45.2% ± 5.1%) and monocyte fractions (18.3% ± 2.4%) alongside reduced CD8^+^ T cells (12.3% ± 3.6%) and NK cells (5.2% ± 1.3%), reflecting a combined state of hyperinflammation and impaired cytotoxic immunity. These quantitative observations provide mechanistic insights into early postoperative mortality and align with prior reports linking elevated IL-6 and CXCL10 levels with adverse outcomes in sepsis and perioperative critical illness, while expanding these findings into the SICU oncology context.

Comparatively, previous studies have demonstrated that isolated clinical parameters or single inflammatory biomarkers yield moderate predictive performance (e.g., APACHE II 30-day mortality prediction accuracy 65%–72%), whereas our integrative approach incorporating both gene expression and immune cell fractions substantially enhances prognostic precision. Moreover, the PIS captures the multidimensional immune state, including innate-adaptive imbalance, which traditional scoring systems fail to quantify. These findings not only corroborate existing concepts of immune exhaustion and dysregulation in critically ill patients but also provide actionable information for targeted interventions, such as IL-6 blockade or adoptive cytotoxic T-cell therapies, by identifying patients with the highest inflammatory burden and lowest cytotoxic potential.

Model Consensus Aggregation (MCA) is a computational aggregation framework designed to integrate model outputs by minimizing the influence of outliers and unstable estimators through consensus-based optimization. In this study, MCA is employed as a robust aggregation baseline for comparative evaluation. Furthermore, we adopted an enhanced variant, enhanced Model Consensus Aggregation (e-MCA), which incorporates adaptive weighting and robustness constraints to improve stability under heterogeneous and adversarial conditions. The e-MCA framework aims to achieve reliable global model performance by emphasizing statistically consistent contributions while suppressing noisy or corrupted updates.

In summary, the combination of high-throughput transcriptomics with computational immune deconvolution offers a robust framework for deriving quantitative, clinically relevant prognostic biomarkers in SICU tumor populations. Our results underscore the potential for precision immunoprofiling to inform risk-adapted management and support the development of targeted immunomodulatory strategies. Further multi-center validation studies and functional analyses of the 12-gene signature are warranted to confirm generalizability and mechanistic relevance, ultimately enabling translation into routine SICU clinical practice.

## Data Availability

The datasets presented in this article are not readily available due to institutional ethical requirements, patient privacy protection obligations, and applicable regulations on human genetic resource data. Requests to access the de-identified clinical datasets should be directed to Chaoyu Wu at wuchaoyu@gmu.edu.cn.
